# A qualitative exploration of healthcare professionals’ perspectives on the role of pharmacists in screening older adults for depression during medication reviews

**DOI:** 10.1007/s11096-026-02171-7

**Published:** 2026-06-18

**Authors:** Duha N. Gide, Sarira El-Den, Lisa Kouladjian O’Donnell, Claire L. O’Reilly

**Affiliations:** 1 Faculty of Medicine and Health, The University of Sydney School of Pharmacy, The University of Sydney, Building Number A15, Science Rd Camperdown, Sydney, NSW Australia; 2 Laboratory of Ageing and Pharmacology, Kolling Institute, Faculty of Medicine and Health, The University of Sydney and the Northern Sydney Local Health District, Sydney, NSW Australia; 3 Present Address: Australian Institute of Health Innovation, Macquarie University, 75 Talavera Road, North Ryde, Sydney, NSW 2109 Australia

**Keywords:** Aged, Depression, Depression screening, Pharmacists, Medication review

## Abstract

**Introduction:**

Late-life depression (LLD) (depression occurring in individuals aged ≥ 65 years) often goes underdiagnosed and undertreated. Pharmacists who conduct medication reviews may play a significant role in LLD screening.

**Aim:**

This study aimed to explore healthcare professionals’ (HCPs) perspectives regarding the role of pharmacists in LLD screening for older adults during medication reviews.

**Method:**

A semi-structured interview guide was developed, guided by the Theoretical Framework of Acceptability (TFA). HCPs across Australia were recruited via purposive sampling through professional organisations’ social media and e-newsletters distributed by primary health networks, and via convenience sampling through departmental meetings and emails at Royal North Shore Hospital, New South Wales, Australia. The interviews explored HCPs’ acceptability and perspectives regarding the role of pharmacists in LLD screening. Key themes and subthemes were identified using inductive thematic analysis. Each subtheme was then deductively mapped to the TFA constructs.

**Results:**

Twenty-eight HCPs participated in the interviews, including 10 credentialed pharmacists (35.7%), nine hospital pharmacists (32.1%), five geriatricians (17.9%) and four general practitioners (14.3%). Most participants were female (n = 22; 78.6%) and practising in New South Wales, Australia (n = 17; 60.7%). The duration of interviews ranged from 21 to 55 min (M = 33 min, SD = 9 min). Five key themes were identified, including Considerations for service delivery, Patients’ homes as screening setting, Resource requirements, Timely follow-up, and Benefits of screening. These themes were further divided into eight subthemes. HCPs generally viewed pharmacist-delivered LLD screening as acceptable, provided certain factors were addressed prior to implementation. Facilitators such as increased patient comfort in their homes and the importance of mental health training were noted; however, barriers such as time constraints, lack of remuneration for additional services and the difficulties of timely follow-up may impede the provision of such services.

**Conclusion:**

HCPs were generally accepting of pharmacist-delivered LLD screening during medication reviews, and perceived pharmacists to have a role in such services. Recommendations for future implementation include targeted mental health training for credentialed pharmacists, appropriate remuneration, and timely follow-up pathways for at-risk patients.

**Supplementary Information:**

The online version contains supplementary material available at https://doi.org/10.1007/s11096-026-02171-7.

## Impact statements

- Healthcare professionals recognise pharmacists’ potential role in late-life depression screening during medication reviews, underscoring the need to address barriers to ensure effective and sustainable service delivery.
- Implementing remuneration models, mental health training initiatives, and establishing clear referral pathways may improve service delivery and increase pharmacists’ identification of older adults at risk of depression.
- Utilising depression screening tools during medication reviews may facilitate timely follow-up, improve mental health outcomes in older adults, and strengthen pharmacists’ contributions to early intervention and improved quality of life for older adults.

## Introduction

Depression has been identified as one of the most common mood disorders in older adults globally [1, 2], affecting approximately 10–20% of those aged 60 years and above [3–5]. Late-life depression (LLD), typically defined as depression occurring in adults aged ≥ 65 years [6], may result in significant distress, functional and cognitive impairment [7], and increased mortality rates [8]. As such, untreated depression can significantly reduce quality of life among older adults aged ≥ 65 years [7].

Various healthcare professionals (HCPs) play an important role in the care of older adults, including physicians, nurses, and pharmacists [9, 10]. An interdisciplinary approach may facilitate effective and efficient person-centred care of older adults [11]. This is particularly important as LLD is often underdiagnosed and undertreated [12]. LLD can be difficult to diagnose due to the association of depressive symptoms with normal consequences of ageing [13] and overlapping symptoms with other medical conditions prevalent in older adults, such as dementia [14]. As pharmacists have a growing role in mental healthcare [15], they may be well-placed to support the timely detection of LLD. A recent mixed-methods pilot study involving 39 community pharmacies demonstrated that pharmacists may have a role in LLD screening in community pharmacy settings, with such services being identified as acceptable and useful by pharmacists and patients [16]. This pilot study also highlighted the importance of targeted training to improve pharmacists’ capability to provide such services [16]. Previous studies have similarly demonstrated pharmacists’ capability to provide depression screening in various settings, such as diabetes care clinics [17], women’s health clinics [18], and hospitals [19], with a recent scoping review supporting the expanding role of pharmacists in depression management [20].

In addition to screening services, pharmacists may have a role in mental healthcare through the provision of pharmaceutical care interventions, such as medication reviews [21]. This notion is supported by previous research, which shows that pharmacists may contribute to a multidisciplinary approach to mental healthcare through medication reviews [22, 23]. In Australia, Home Medicines Reviews (HMRs), conducted in patients’ homes, and Residential Medication Management Reviews (RMMRs), conducted in residential aged care facilities, are medication review programs [23] which aim to reduce issues arising from the inappropriate use of medicines, and involve the collaboration of physicians, pharmacists and patients [23–25]. During medication reviews, credentialed pharmacists (i.e., specially trained pharmacists who have received post-registration certification in medication reviews) conduct comprehensive interviews with patients to explore their medications, medical conditions, and overall wellbeing [23]. Following this, they provide the referring physician (e.g., general practitioner (GP) or geriatrician) with a written report detailing their findings and recommendations [23, 26]. Thus, as medication reviews are conducted in patients’ homes or aged care facilities, and cover various aspects of patients’ health, this may ideally place credentialed pharmacists to provide LLD screening.

To gauge the feasibility of pharmacist-delivered LLD screening during medication reviews, it is imperative to first explore HCPs’ acceptability and perspectives regarding such services, particularly those involved in the care of older adults. Acceptability refers to stakeholders’ (e.g., HCPs) perception that a new service is agreeable [27]. Exploring acceptability is necessary to ensure adoption and sustainability of screening interventions [27, 28]. HCPs in this setting include GPs and geriatricians, who liaise with credentialed pharmacists during the medication review process; credentialed pharmacists, who play a key role in ensuring continuity of care for older patients through the provision of medication reviews and by collaborating with GPs [23]; and hospital pharmacists, who play an important role in the care of older patients in transitions of care (to aged care homes or community settings) [29].

## Aim

The aim of this study was to explore HCPs’ (i.e., credentialed pharmacists, hospital pharmacists, GPs and geriatricians) perspectives regarding the role of pharmacists in depression screening for older adults during medication reviews (i.e., HMRs and RMMRs).

## Method

### Study design

A qualitative study consisting of semi-structured interviews explored HCPs’ (i.e., credentialed pharmacists, hospital pharmacists, GPs and geriatricians) perspectives regarding the role of pharmacists in LLD screening during medication reviews.

### Development of study instrument

The Theoretical Framework of Acceptability [30] (TFA; Table 1) was used to inform the development of a semi-structured interview guide which explored HCPs’ perspectives regarding pharmacist-delivered LLD screening during medication reviews (Online Resource 1). The TFA is a framework which explores the appropriateness of healthcare interventions from the perspectives of individuals involved in the service. It considers an individual’s cognitive and emotional response to an intervention and can be used to evaluate the acceptability of new and hypothetical interventions [30].

**Table 1:** The theoretical framework of acceptability (TFA) constructs and definitions [30], with example interview questions^a^

TFA constructs	Definition of constructs	Example interview questions
Affective Attitude	“How an individual feels about the intervention”	What is your opinion of depression screening for older adults by pharmacists during medication reviews? Do you think it would be useful for pharmacists to screen for depression during medication reviews? Are pharmacists well-placed to provide depression screening for older adults during medication reviews?
Burden	“The perceived amount of effort that is required to participate in the intervention”	Do you think older adults would feel comfortable discussing their mental health during a medication review? What kind of training is required to ensure pharmacists are well-equipped to provide such services? What would be an appropriate/feasible length of time for the screening?
Ethicality	“The extent to which the intervention has good fit with an individual’s value system”	Do patients often talk to you about their mental health during medication reviews? Are patients’ mental health problems a common concern you encounter during medication reviews? What may be the implications of depression screening during medication reviews for pharmacists?
Intervention coherence	“The extent to which the participant understands the intervention and how it works”	Do you think older patients’ homes are an appropriate setting to conduct depression screening? How would you ensure older adults receive the appropriate care following screening?
Opportunity costs	“The extent to which benefits, profits or values must be given up to engage in an intervention”	In your opinion, what factors would deter older adults from participating in such a service? What would prevent pharmacists from delivering such a service? What are your thoughts on the time it would take to provide the screening? Would you expect an incentive/remuneration to deliver such a service?
Perceived effectiveness	“The extent to which the intervention is perceived as likely to achieve its purpose”	What may be the outcomes for LLD screening during medication reviews? What factors would encourage an older adult to engage in depression screening during medication reviews? What factors would prompt a pharmacist to recognise the need for a depression screening service during medication reviews?
Self-efficacy	“The participant's confidence that they can perform the behaviour(s) required to participate in the intervention”	How confident would you feel screening older adults for depression while performing medication reviews? How comfortable would you feel screening older adults for depression during medication reviews? Do you believe you have the necessary skills to effectively deliver these services?

^a^The interview guide was adapted for each healthcare professional group, with certain questions being modified or omitted to align with participants’ professional roles

### Data collection

Credentialed pharmacists, hospital pharmacists and GPs practising in Australia were recruited for the study via purposive sampling. Advanced Pharmacy Australia, the national professional body representing hospital pharmacists, and the Pharmaceutical Society of Australia (PSA), the peak professional organisation supporting pharmacists across Australia, promoted the study to credentialed and hospital pharmacists via social media. In addition, the study was promoted through relevant pharmacist social media groups, primary health networks in Western and Northern Sydney, and The University of Sydney Medical School to recruit GPs. Participants were purposively recruited across different clinical settings and professional roles to capture a diverse range of perspectives. Geriatricians practising at Royal North Shore Hospital in New South Wales (NSW), Australia were recruited using convenience sampling. Geriatricians were informed of the study at a departmental meeting and via emails from the Head of the Department of Aged Care.

HCPs who were interested in participating contacted a research team member (COR and/or DG), after which they were provided with a participant information statement and consent form. Written consent was collected prior to the interview via email, and verbal consent was obtained during the interview. All interviews were conducted by a PhD candidate (DG), who had experience in conducting qualitative interviews. Interviews took place via telephone and were audio-recorded with participants’ consent. Participants were offered the option to review their interview transcript for accuracy; however, no participants requested this. Participants were also remunerated for their time with a $100 gift card. Interviews were conducted from October 2023 to March 2024, at which point no new themes were identified and recruitment concluded.

### Data analysis

All interviews were de-identified by DG and provided codes based on profession (i.e., credentialed pharmacists ‘CP’, hospital pharmacists ‘HP’, geriatricians ‘G’, general practitioners ‘GP’). Interviews were transcribed verbatim and checked for accuracy by DG. Data was thematically analysed in line with Braun and Clarke’s thematic analysis method [31] using an inductive approach. DG reviewed transcripts several times to gain familiarity with the content. Transcripts were then imported into NVivo 12 software [32] for coding. Codes were generated by identifying recurring patterns of data. All coding was performed by DG; however, COR also independently coded approximately 20% of transcripts to reduce bias and improve reliability of the analysis. DG and COR compared and discussed codes, after which similar codes were grouped together to form candidate themes. Candidate themes were divided into subthemes, both of which were reviewed and refined by all four authors until all themes and subthemes were agreed upon. Subthemes were then linked with supporting data (i.e., illustrative quotes). Following this, each subtheme was deductively mapped to the TFA [30], with some subthemes being mapped to multiple TFA constructs. Mapping to the TFA was reviewed and discussed by all authors until consensus was reached. The presentation of data was guided by the COnsolidated criteria for REporting Qualitative studies (COREQ): 32-item checklist [33].

### Ethics approval

This study received ethical (2023/ETH00691) and governance (2023/STE01140) approval from the Northern Sydney Local Health District (NSLHD) Human Research Ethics Committee in NSW, Australia.

## Results

Twenty-eight HCPs participated in the interviews. Ten HCPs stated that their primary professions were credentialed pharmacists (35.7%), nine hospital pharmacists 32.1%), five geriatricians (17.9%) and four GPs 14.3%). The duration of interviews ranged from 21 to 55 min (M = 33 min, SD = 9 min). The majority of participants were female (n = 22) and practising in NSW (n = 17) (Table 2).

**Table 2:** Characteristics of healthcare professional groups

Participant Characteristics	Credentialed pharmacists (n = 10)	Hospital pharmacists (n = 9)	Geriatricians (n = 5)	General practitioners (n = 4)	Total (n = 28)
Female	8	6	4	4	22
*State*					
New South Wales Queensland Victoria Other	3 3 2 2	5 0 2 2	5 0 0 0	4 0 0 0	17 3 4 4

Five themes were identified, three of which were further divided into eight subthemes. Themes and subthemes were independently mapped to the following TFA construct(s): perceived effectiveness (n = 7), affective attitude (n = 5), intervention coherence (n = 4), burden (n = 2), ethicality (n = 2), self-efficacy (n = 1), and opportunity costs (n = 1).

### Considerations for service delivery

#### Routine vs. targeted screening

GPs and geriatricians noted that offering LLD screening to all older adult patients may increase the identification of those at-risk of depression and improve engagement with the service *(intervention coherence)*; “*If it was made like a generic, “this is what we do as part of the medication review”… that would encourage them*” (GP3).

However, some participants believed that screening should be targeted towards at-risk patients, as it may not be necessary or appropriate to introduce screening to patients purely because of their age *(intervention coherence); “I don't think it's necessary to screen absolutely everyone*” (G5).

#### Screening is unexpected

It was noted that incorporating depression screening into medication reviews may confuse patients and GPs, given the expectation that these reviews focus on patients’ medications (*affective attitude)*; *“I would be a bit like “ohh, why did you do this?” And that's not really what I sent them for*” (GP2).

This may affect patients’ likelihood to engage in the screening service (*perceived effectiveness)*; “*If the question came out of the blue with the review and the patient was expecting someone to go through their medications, some may or may not respond as well*” (G4).

#### Cognitive testing prior to LLD screening

Cognitive testing and depression screening were said to go hand in hand due to the association between depression and cognitive decline. As such, participants highlighted the importance of performing cognitive screening prior to depression screening *(intervention coherence)* to provide a more comprehensive assessment of patients’ mental health *(perceived effectiveness); “I don’t think it’s possible to screen for depression without doing a cognitive screen.”* (GP4).

#### Pharmacist-patient relationship

Participants also noted that medication reviews may be conducted by pharmacists who are unfamiliar to the patient, affecting patients’ comfort and willingness to open up about their mental health *(affective attitude); “They don’t necessarily know the pharmacist well, as opposed to hopefully their GP, so might be less comfortable to disclose things*” (GP3).

#### Perspectives on the need for LLD screening

GPs expressed that if they were concerned about a patients’ mental health, they would screen the patient themselves *(affective attitude)*; “*If I wanted to do screening, I would just do it myself*” (GP1).

Furthermore, geriatricians noted that patients referred for medication reviews are generally already linked in with the healthcare system and monitored by their physician, suggesting limited need for these services *(perceived effectiveness); “These are patients who have been referred by healthcare providers, so they’re already people who* [are] *already engaged with healthcare”* (G2).

GPs felt that depression screening during medication reviews may optimise patients’ health outcomes and help at-risk individuals receive appropriate care *(ethicality)*; “*It's a really common problem, so anything that we can do to try and identify it earlier would be very beneficial*” (GP1).

### Patients’ homes as screening setting

Pharmacists noted that incorporating depression screening into medication reviews may be facilitated by pharmacists already being in patients’ homes to discuss their health. This may encourage patients to be more open to talking about their mental health *(intervention coherence* and *perceived effectiveness)*; “*People have already opened their doors for us to enter their homes, opened their hearts to, you know… talk about their conditions*” (CP10).

Additionally, it was reported that the state of patients’ homes may reflect their mental wellbeing, and hence prompt pharmacists to initiate depression screening *(intervention coherence* and *perceived effectiveness)*; “Y*ou get an idea of how they're looking after yourself* [themselves] *from the condition of the house*” (CP3).

Patients were also thought to be more comfortable and willing to discuss their mental health in their homes, as opposed to an environment they are unfamiliar with *(perceived effectiveness)*; “*They feel more comfortable in their familiar environments…so they might be more willing to open up*” (HP2).

However, participants flagged that a potential lack of privacy in patients’ homes due to the presence of other family members or carers may deter patients from engaging in such services *(perceived effectiveness)*; “*Some people are better doing it when they've got no family around and they're more likely to open up because they don't want their family to know that they're struggling*” (CP1).

### Resource requirements

#### Time management

Participants mentioned there was already a lot of information to cover in medication reviews, and interview fatigue may affect patients’ engagement *(burden)*; “*Getting fatigued, if the questions are going on too long, like they’ve gone through a list of 20 medications and then “let's talk about whether or not you have depression or not”…and not want to engage with the pharmacist*” (G5).

Additionally, geriatricians noted pharmacists’ may be hesitant to provide depression screening during medication reviews, as adding this service may limit the number of reviews pharmacists can complete *(opportunity costs)*; “*It may affect their workflow and how many they can see or review in a day*” (G4).

However, as there is no time limit for medication reviews, this may allow for more comprehensive assessments of patients’ mental health *(intervention coherence* and *perceived effectiveness)*; “*If we optimise the time we spend with them, we can actually pick things up that maybe other members of the health care team, who don't necessarily have the same time we have*, [don’t]” (HP6).

#### Need for remuneration

Participants mentioned that additional remuneration for providing depression screening during medication reviews may increase pharmacists’ willingness to provide such services, particularly considering pharmacists may feel undervalued when providing services free of charge *(burden)*; “*On top of having to do my medication review plus other services and not getting compensated, sometimes it's really hard for us to continue doing what we're doing*” (CP2).

#### Need for training

Training was said to be imperative to ensure pharmacists can respond appropriately to patients in distress, such as if a patient were to experience a mental health crisis *(perceived effectiveness)*; “*I definitely think Mental Health First Aid training would be an essential part because… if they do start to exhibit like severe signs of suicidal ideation, that's a medical emergency*” (HP5).

Training may also improve pharmacists’ confidence to provide *screening (self-efficacy)*; “*It's actually quite a niche unit area that the majority of pharmacists are not trained to do, so it's understandable that we do not have the confidence to actually start this conversation and talk about it*” (HP1).

### Timely follow-up

Participants perceived the usefulness of such services as being contingent upon the provision of follow-up care *(perceived effectiveness)*; “*Screening would only be useful if there can be some action that can be taken beyond that*” (G4).

GPs and geriatricians noted that pharmacists may experience difficulties informing GPs of screening results, as it may be difficult for pharmacists to contact GPs *(perceived effectiveness)*; “*When it's included sort of just as a letter to the GP, it isn't always followed up on appropriately… Unless it's communicated verbally, it's probably not always going to get followed up… getting through to a GP is not always the easiest*” (G2).

### Benefits of screening

#### Screening alerts medical team to mental health problems

Depression screening during medication reviews was also said to add value to the healthcare team by allowing for a more robust overview of patients’ health, potentially facilitating earlier diagnosis and treatment for patients *(affective attitude* and *ethicality)*; “*If we can assist the team in that manner and pick things up like depression… it would also add value to the healthcare team and also would potentially mean that patients will be treated earlier*” (HP6).

#### Combatting mental health stigma

Participants also mentioned that these screening services may allow pharmacists to screen patients who would otherwise not seek help for their mental health due to stigma *(ethicality)*; “*When they have like mental health conditions, they feel like they can manage it on their own… there's a lot of sometimes stigma in receiving care*” (HP4).

Participants also emphasised that depression screening during medication reviews may provide an opportunity for pharmacists to educate patients regarding the fact that depression is not a normal part of ageing, encouraging patients to seek help for their mental health *(ethicality)*; “*In the older generation, they tend to think it's just, you know, the process of ageing… without screening, we won’t know that people are suffering in silence*” (CP10).

#### Increased job satisfaction and skills

Pharmacists noted that providing depression screening during medication reviews may result in increased job satisfaction due to their role in improving patients’ health outcomes *(affective attitude)*; “*It makes me feel good, because I've done a rewarding thing*” (HP1).

## Discussion

This study explored the potential role of pharmacists in screening older adults for depression during medication reviews. Findings indicate that while such services may be useful in supporting the early detection and treatment of LLD, several aspects of this potential service need to be considered prior to implementation. This includes screening setting, the need for cognitive testing prior to screening, availability of resources and the importance of follow-up procedures to ensure patient safety and continuity of care.

Participants highlighted that patients would be more willing to disclose information pertaining to their mental health in their homes, as opposed to environments they are less comfortable in, such as physicians’ offices or community pharmacies. This may, in turn, affect the effectiveness of pharmacist-delivered LLD screening during medication reviews. Community pharmacists reported that a lack of privacy may impede older adults’ willingness to participate in LLD screening in community pharmacies [34]. However, as noted in the current study, there may be a potential lack of privacy from other family members or carers being in the home, impacting the effectiveness of such services by hindering older adults’ engagement. This is supported by Shen et al., who noted that privacy is especially important to consider during mental health conversations with both adults and older adults due to the stigma associated with mental health [35]. Interestingly, White et al., who explored HMR recipients’ perspectives of HMRs, noted that none of the recipients reported any home privacy concerns [25]. They also found patients’ homes to be an ideal place to discuss their health as they felt more comfortable doing so in their homes, as opposed to in a community pharmacy [25]. Thus, patients’ homes may be suitable settings for pharmacists to provide depression screening for older adults, compared to a community pharmacy.

Participants also noted that as depression screening is not the focus of medication reviews, it may be difficult to incorporate such services due to time constraints, increasing the burden for pharmacists. As shown in a study by Patounas et al., which explored credentialed pharmacists’ time investments during the HMR process, 70.2% of pharmacists (n = 179) reported that it took them 30–60 min to conduct the medication review interview with patients [36]. Czarniak et al., also reported that, based on a survey completed by 178 credentialed pharmacists, the entire medication review process, from receiving the referral, to conducting the interview, and preparing the report for GPs’ consideration, may take an average of 3 h for HMRs and 2 h for RMMRs [37]. Thus, while pharmacists are afforded unlimited time to conduct medication reviews, adding a depression screening service to medication reviews would mean pharmacists are able to complete less medication reviews in a day, affecting their remuneration. As lack of remuneration has been identified as a barrier to the provision of pharmacy services [38, 39], this may impact pharmacists’ willingness to provide such services.

Participants expressed that age-associated cognitive decline may pose potential challenges to the effectiveness of pharmacist-delivered LLD screening during medication reviews. Thus, they proposed cognitive testing be provided to patients prior to LLD screening. Cognitive function declines (to varying degrees) due to the natural process of ageing [40]. Cognitive deficits have been associated with depression, with symptoms such as memory dysfunction being identified in patients at-risk of depression [41]. Overlapping symptoms between cognitive disorders (e.g., dementia) and depression have also been reported, such as sleep disturbances, difficulty concentrating and decreased energy [42], making the diagnosis of depression difficult [14]. As such, the provision of cognitive testing prior to LLD screening may not only ensure patients are capable of answering screening questions, but also allows for a more comprehensive overview of patients’ health, particularly considering the linkage between cognitive decline and depression. Community pharmacists have previously demonstrated their capability to provide cognitive testing and referrals for patients [43]. Abed et al. also investigated the use of cognitive testing tools during medication reviews and found that such services improve patients’ health outcomes [44]. Thus, it may be useful to provide cognitive testing to patients, followed by LLD screening. This is supported by GPs in the current study, who highlighted that depression screening should not be conducted in isolation, but rather alongside other cognitive and mental health assessments. However, some GPs and geriatricians indicated a preference to conduct screening themselves, highlighting variability in HCPs’ views. Alternatively, credentialed pharmacists may check whether a recent cognitive screen was conducted, such as by contacting the patient’s GP or reviewing hospital discharge summaries, before conducting the HMR. This may help alleviate the increased time it would take to provide cognitive screening in addition to LLD screening.

The importance of timely post-screening follow-up procedures was emphasised by participants. It was noted that if screening results were to solely be included in pharmacists’ medication review reports provided to GPs, this may result in a potential delay in the care of patients at-risk of LLD. As reported by Ahn et al., there is often a delay in the time GPs receive pharmacists’ medication review reports, and when they next see their patients to discuss results [45]. Considering the potential need for urgent action for some patients, this delay may prevent patients from receiving mental healthcare in a timely manner. To combat this, participants in the current study suggested communicating screening results with physicians via phone call. This aligns with guidance from the PSA which recommends that credentialed pharmacists verbally communicate critical matters to GPs [46]. Hence, to ensure patients receive appropriate care in a timely manner, there is a need for pharmacists to work around these system challenges and also be capable of providing support to patients in the meantime. This may be achieved through mental health training, such as MENTAL HEALTH FIRST AID®(MHFA®) [47], which has been shown to improve pharmacists’ comfort, confidence and skills to provide mental healthcare [48, 49], and increase first aid actions towards people experiencing mental health problems [50]. This may not only improve their self-efficacy, but also reduce the risk of negative impacts on pharmacists’ mental health when managing challenging cases or taking on additional responsibilities [49]. However, it should be noted that MHFA® training does not provide guidance on actions to take based on screening results, highlighting the need for more than just MHFA® training in this context. Continuity of care may also be ensured through the establishment of integrated care pathways, which are usually multidisciplinary, patient-centred pathways of care which cover all phases of a patient’s journey [51, 52].

### Strengths and limitations

To our knowledge, this is the first study to explore the role of pharmacists in providing depression screening for older adults during medication reviews. This study explored the perspectives of HCPs who are directly involved in the medication reviews process and/or in the care of older adults, including credentialed pharmacists, hospital pharmacists, GPs and geriatricians, allowing for a robust exploration of views towards such potential services. This study also employed the use of the TFA in the development of the interview guide and as part of data analysis. This provided an evidence-based guide for assessing the acceptability of healthcare interventions, thus strengthening study findings and supporting future research by identifying factors that may support the implementation of such services [53]. Additionally, credibility was supported by triangulating perspectives across HCP groups and peer debriefing, while dependability and confirmability were ensured through detailed records of interviews, coding, and analytic decisions. However, there were also limitations associated with this study. Firstly, author DG, who conducted the interviews and data analysis, is a pharmacist and it is possible that her experience and knowledge regarding the pharmacy profession and pharmacists’ roles in mental healthcare may have influenced the analysis of collected data. However, frequent discussions were had between all authors during the data analysis process, and all authors ensured consensus was reached on themes, subthemes and TFA mapping, in order to minimise this potential influence. Remuneration may have also led to social desirability bias; however, maintaining confidentiality of participants’ responses likely reduced this bias. In addition, the perspectives of older adults and community pharmacists were not explored in this study, which would be important for assessing the acceptability of such services. Furthermore, only geriatricians practising in NSW and credentialed pharmacists, hospital pharmacists and GPs practising in Australia participated in this study. Thus, the perspectives explored in this study may not be representative of HCPs involved in similar medication review programs outside of NSW or Australia. Furthermore, in order to best target geriatricians for this study, convenience sampling methods were utilised through Royal North Shore Hospital in Sydney, Australia. However, it is acknowledged that this strategy differed to the purposive sampling methods used in the recruitment of other HCP groups.

## Conclusion

This study demonstrated that HCPs (credentialed pharmacists, hospital pharmacists, GPs and geriatricians) generally perceived pharmacists to have a potential role in LLD screening during medication reviews, with such services being viewed as useful in supporting the early identification and treatment of LLD. While unlimited time and the privacy of patients’ homes may facilitate the provision of such services, for the effective provision of such services, several barriers need to be addressed. In particular, participants emphasised the need for various barriers to be addressed for the effective delivery of these services, such as time constraints, lack of remuneration, the need for cognitive testing prior to depression screening, and the importance of follow-up procedures. Future studies may investigate older adults’ and community pharmacists’ perspectives on the role of pharmacists in such services and explore the development of guidelines to ensure patients are appropriately followed-up post-screening.

## Supplementary Information

Below is the link to the electronic supplementary material.

Supplementary file1 (DOCX 16 kb)

## Data Availability

No datasets were generated or analysed during the current study.
