# Supplementary material for: A qualitative exploration of healthcare professionals’ perspectives on the role of pharmacists in screening older adults for depression during medication reviews
Source: Int J Clin Pharm. 2026 Jun 18;48(5):1937–46. doi: 10.1007/s11096-026-02171-7 (PMC13569508; doi:10.1007/s11096-026-02171-7)
Supplement: Supplementary file 1 [file 11096_2026_2171_MOESM1_ESM.docx]

**A qualitative exploration of healthcare professionals’ perspectives on the role of pharmacists in screening older adults for depression during medication reviews**

**International Journal of Clinical Pharmacy**

Duha N. Gide, Sarira El-Den, Lisa Kouladjian O’Donnell, Claire L. O’Reilly

Corresponding author: Duha N. Gide*

Sydney Pharmacy School, The University of Sydney, Building Number A15, Science Rd Camperdown, NSW, 2006, Australia

Ph: +61 2 9351 7114

E: [duha.gide@sydney.edu.au](mailto:duha.gide@sydney.edu.au)

**Supplementary Materials**

**Online Resource 1: INTERVIEW GUIDE**

*(Note 1: This interview guide has been guided and structured according to the seven domains of the Theoretical Framework of Acceptability).*

*(Note 2: This interview guide has been worded specifically for credentialed pharmacists’ interviews; however, wording will vary based on the healthcare professional being interviewed).*

**Affective attitude (how individual feels about intervention):**

**-** Do you think there is a need for older adults to be screened for depression?

- What is your opinion regarding depression screening for older adults by pharmacists during medication reviews?

- Do you think it would be useful for pharmacists to screen for depression during medication reviews? *If yes, why? If no, why not?*

- Are pharmacists well-placed to provide depression screening for older adults during medication reviews? *If yes, why? If no, why not?*

**Burden (perceived effort required to participate in the intervention):**

- Do you think older adults would feel comfortable discussing their mental health during a medication review? *If yes, why? If no, why not?*

- What kind of training is required to ensure pharmacists are well-equipped to screen older adults for depression during medication reviews?

- What would be an appropriate/feasible length of time for the screening?

**Perceived effectiveness (the extent to which an intervention is perceived as likely to achieve its purpose):**

- What may be the outcomes for LLD screening during medication reviews? *What may be the benefits or harms of LLD screening?*

- In your opinion, what factors would encourage an older adult to engage in depression screening during medication reviews?

- What factors would prompt a pharmacist to recognise the need for a depression screening service during medication reviews?

**Intervention coherence (the extent to which the participant understands the intervention and how it works):**

- At what point during the medication review process would late life depression screening be best incorporated?

- Do you think older patients’ homes are an appropriate setting to conduct depression screening?

- What are your expectations regarding a late life depression screening tool which may be used during medication reviews?

- How would you ensure older adults receive the appropriate care following screening?

**Opportunity costs (the extent to which benefits, profits or values must be given up to engage in the intervention):**

- In your opinion, what factors would deter older adults from participating in such a service?

- What would prevent pharmacists from delivering such a service?

- What are your thoughts on the time it would take to provide the screening?

- If for example, a depression screening service was designed and it took approximately 20-30 minutes, in addition to the time normally taken to complete a medication review. What kind of support would encourage you to provide this service? Would you expect an incentive/remuneration to deliver such a service? Do you think current reimbursement for medication reviews would be enough to cover the additional service? Would reimbursement affect your willingness to provide screening?

**Self-efficacy (the participant’s confidence that they can perform the actions required to participate in the intervention):**

- How confident would you feel screening older adults for depression while performing medication reviews?

- How comfortable would you feel screening older adults for depression while performing medication reviews?

- Do you believe you have the necessary skills to effectively deliver these services? *Why/why not?*

**Ethicality (the extent to which the intervention was a good fit with the individual’s value system):**

- Do patients often talk to you about their mental health during medication reviews? *If so, could you tell me a little bit about what they share?*

- Are patients’ mental health problems a common concern you encounter during medication reviews? *If so, could you elaborate on this?*

- What may be the implications of depression screening during medication reviews for pharmacists?
